# Transport Time Does Not Substantially Alter RNA Expression in Human Ovarian Tissue After Standardized Slow-Freezing for Fertility Preservation

**DOI:** 10.3390/jcm15093260

**Published:** 2026-04-24

**Authors:** Iwona Scheliga, Jana Bender-Liebenthron, Jan-Steffen Kruessel, Alexandra Knebel, Dunja M. Baston-Buest, Alexandra P. Bielfeld

**Affiliations:** Department of OB/GYN and Reproductive Medicine, UniKiD & UniCareD, Medical Faculty and University Hospital Duesseldorf, Heinrich-Heine University Duesseldorf, 40255 Duesseldorf, Germanyalexandra.knebel@med.uni-duesseldorf.de (A.K.);

**Keywords:** ovarian tissue overnight transport, ovarian tissue cryopreservation, oncofertility, female fertility, FertiPROTEKT, RNA-seq

## Abstract

**Background:** Fertility preservation aims to maintain reproductive potential in patients undergoing potentially gonadotoxic treatments, increasingly relying on centralized cryobanks requiring ovarian tissue transport. Ovarian tissue cryopreservation is a widely implemented, evidence-based procedure for young women (age 18–35) with a regular ovarian reserve. The ovaries of patients are typically transported overnight to a centralized cryobank for freezing and storage, using a certified hypothermic organ preservation solution such as histidine-tryptophan-ketoglutarate (HTK) at 4–8 °C. The molecular effects of transport on ovarian tissue remain unclear. **Methods**: In this prospective study of 36 breast cancer patients, we compared whole-transcriptome RNA (RNA-seq) expression in 18 frozen–thawed ovarian biopsies after overnight hypothermic transport followed by slow-freezing versus 18 direct slow-freezing within ≤2 h under FertiPROTEKT-standard conditions. **Results**: The RNA-seq analysis identified 6 significantly upregulated genes (Bonferroni < 0.05, fold change > 1.5), including histone H2B and mitochondrial NADH dehydrogenase subunit 6 (MT-ND6). The small number of differentially expressed genes suggests only limited transcriptional changes between the two transport conditions. H2B upregulation was confirmed by qPCR, while MT-ND6 showed only moderate levels in RNA-seq but remained stable in qPCR. Immunohistochemical analysis confirmed protein presence and localization in formalin-fixed tissue from four samples, constituting, to our knowledge, the first report of MT-ND6 protein expression in human ovarian tissue. **Conclusions**: Overall, these results are consistent with subtle changes in chromatin organization and mitochondrial energy metabolism. Since RNA-seq revealed only modest differences in gene expression, with no appreciable up- or downregulation of apoptosis- or damage-related genes after ≤24 h, this indicates tissue stability under the studied combined conditions (transport + cryopreservation). These findings are consistent with the feasibility of the workflow under the studied conditions of centralized ovarian tissue cryobanking combined with overnight transportation and hypothermic HTK solution.

## 1. Introduction

Fertility preservation is becoming increasingly important in reproductive medicine due to the rising number of affected patients of reproductive age worldwide [1]. For almost two decades, fertile patients who have had to undergo potentially gonadotoxic treatments have been able to preserve their reproductive potential [2]. Besides cryopreservation of sperm and oocytes, female patients may also choose ovarian tissue cryopreservation (OTC) by vitrification or slow freezing [3]. The advantage of freezing ovarian tissue (OT) is that it can be done without hormonal stimulation and is particularly considered for patients with limited time to start their gonadotoxic treatment, such as chemotherapy [4]. Per ESHRE, OTC complements oocyte cryopreservation in young patients with sufficient reserve facing gonadotoxic therapy (time pressure/contraindications) [5]. 

The ovary, or half of it, is removed by laparoscopy and has to be transported to a cryobank that has expertise in ovarian tissue cryopreservation (OTC) [6]. Clinical OTC outcomes show LBR ~26–28% per patient regardless of transport [7,8,9]. In the FertiPROTEKT network, the transport and cryopreservation of ovarian tissue are predominantly carried out by three centralized cryobank institutions, all of which use a standardized overnight transport protocol and slow-freezing protocol [8,9,10,11]. This workflow reduces local processing variations within the FertiPROTEKT network.

However, all of these methods share the common requirement that, to maintain tissue viability and integrity, the ovarian tissue must be placed in a protective solution under stable cooling conditions (4–8 °C) within a sealed container [12].

As a protective solution, various media can be used for ovarian tissue transport, including cell culture media, commercial transport media, self-prepared media, and organ preservation solutions [13,14]. Histidine-Tryptophan-Ketoglutarate (HTK) is widely used for clinical organ preservation and has been shown to maintain organ function, for example, in hearts and kidneys, until transplantation [15]. Its composition, including histidine for pH stabilization, tryptophan for membrane protection, ketoglutarate as an energy source during ischemia, and electrolytes to prevent cellular edema, helps buffer acidosis, reduce oxidative stress, and lower metabolic activity while maintaining minimal cellular function [16].

This protective solution is further used during ovarian tissue processing, where the cortex is processed into cortical strips and cryopreserved using standard protocols [8,10,11]. Once frozen in the vapor phase of liquid nitrogen, storage conditions remain highly stable, and extended cryostorage appears safe; however, data on very long storage durations for ovarian tissue remain limited [17]. Generally, several years after gonadotoxic treatment, the disease-free female patient decides to undergo transplantation when she fails to become spontaneously pregnant [18].

After about 1 week following orthotopic or heterotopic transplantation, the neovascularization and reperfusion of the follicles begin, and menstrual cycles can often be restored [7,19]. Recent transplantation data from the FertiPROTEKT network have shown that pregnancy and live birth rates are comparable for patients who received an ovarian autograft after overnight transport (ONT) compared to patients whose ovaries were cryopreserved on the day of retrieval (DF) [9].

Although it is known that the HTK solution maintains tissue integrity and quality of whole organs, processes within ovarian tissue on a molecular level are still unknown, and it remains unclear whether there is a positive influence of ONT on molecular characteristics in the ovarian tissue before and after cryopreservation. Despite comparable pregnancy and live birth rates after ONT and DF in clinical cohorts, this study aimed to reveal possible differentially expressed genes after incubation in HTK solution for 24 h compared to processing immediately after retrieval of the OT.

## 2. Materials and Methods

### 2.1. Patient Recruitment and Study Approval

In this prospective study, ovarian cortex biopsies were obtained from 36 patients diagnosed with breast cancer between 20 and 40 years of age (mean age: 30.4 ± 3.8). The anti-Müllerian hormone (AMH) levels were measured, with a mean value of 3.1 ± 1.6 ng/mL, and the follicular density was determined, yielding an average of 75.4 ± 59.6 follicles per 3 × 2 mm biopsy (see Supplementary Figure S1). Samples from clinics with on-site cryobank underwent direct freezing (DF, *n* = 18, ≤2 h post-retrieval); samples from distant clinics were transported overnight to UniCareD Cryobank (ONT, *n* = 18, 17–24 h, ≤24 h in HTK at 4–8 °C). Frozen-thawed biopsies were obtained from UniSaFeD Biobank (protocol 2024-2781-bio). Ovarian tissue was collected and cryopreserved at the University Hospital Duesseldorf from November 2024 till September 2025 under routine clinical conditions. Upon arrival, the temperature of the transport containers was confirmed to be within 4–8 °C by an integrated data logger. Throughout the entire recruitment period of this study, the same modified Gosden slow-freezing protocol recommended within the FertiPROTEKT network was consistently applied to all samples, without deliberate changes to cryoprotectant composition or the overall freezing/thawing scheme [9]. The study was approved by the University of Duesseldorf Ethics Committee (protocol 2025-3548). All patients provided written informed consent, and all procedures were conducted in accordance with institutional and national ethical guidelines.

### 2.2. Cryopreservation of Ovarian Tissue

The preparation of the ovary involves the removal of stromal tissue, resulting in a thin layer of cortex. This layer is then processed into several equal strips (8 × 4 × 2 mm) within a 90 mm dish (Nunc Thermo, Thermo Scientific, Waltham, MA, USA) containing 20 mL of HTK solution, placed on a cooling plate (Fryka, ULK 602, Esslingen, Germany). This procedure is conducted under a laminar flow hood (Herasafe KS, Thermo Scientific, Waltham, MA, USA). 3 × 2 mm ovarian biopsies were obtained using a biopsy needle punch (PFM Medical GmbH, Cologne, Germany). The biopsies and the cortical strips are equilibrated in Multipurpose Handling Medium (MHM)-Complete (Nexpring Health, formerly Irvine Scientific, Santa Ana, CA, USA) containing 10% DMSO (WAK-Chemie Medical GmbH, Steinbach, Germany) as a cryoprotectant for 40 min at 4–8 °C. They are then placed in 1.8 mL of cryomedium in 2 mL cryotubes (Thermo Scientific, Waltham, MA, USA). Cryopreservation is achieved through slow freezing using a programmable freezer (Icecubes 14S, SY-LAB Geräte GmbH, Neupurkersdorf, Austria), which gradually cools the samples at −2 °C/min to −6 °C (automatic seeding), holds the temperature for 5–8 min, and then continues cooling at −0.3 °C/min to −40 °C and −10 °C/min to −140 °C. Following this, the samples are stored in the vapor phase of an LN_2_-cryotank, which is equipped with a monitoring and auto-fill control system, at a minimum temperature of −190 °C (MVE CryoSystem 6000 Full Auto, MVE Biological Solutions, Ball Ground, GA, USA). The vapor-phase temperature is routinely maintained between approximately −185 °C and −195 °C, as verified by regular manual measurements. Liquid nitrogen levels are monitored continuously by the tank control unit, and an external 24/7 alarm system alerts staff in case of level deviations. Tanks are opened only briefly for sample handling, and refilling is performed according to a standardized schedule to minimize temperature fluctuations. Cryostorage durations ranged from short- (1 week) to intermediate-term (11 months), within the usual clinical spectrum of our cryobank.

### 2.3. Thawing of Ovarian Tissue

To thaw the biopsies, the cryotube was removed from LN_2_ storage and incubated at room temperature (RT) for 30 s. The tubes were then transferred to a water bath (Grant Instruments, Shepreth, Cambridgeshire, UK) set at 37 °C for 120 s. To remove the cryoprotectant DMSO and rehydrate the cells, the biopsies were placed in decreasing concentrations of sucrose solutions (Sigma-Aldrich, St. Louis, MO, USA) of 0.75 M, 0.375 M, and 0.125 M in Dulbecco’s phosphate-buffered saline (DPBS). Each solution was incubated for 15 min in a 4-well plate (Nunc, Thermo Scientific, Waltham, MA, USA) on a shaker (Grant Instruments, Shepreth, Cambridgeshire, UK) at RT under a sterile workbench (IVF Tech, Gynemed GmbH & Co. KG, Sierksdorf, Germany) operating at 200 cycles per minute. The final step involved incubating the biopsies in 1 mL of DPBS with HSA for an additional washing step for 15 min. Following the thawing process, the biopsies were collected in tubes containing 100 µL of RNAlater™ Solution (Thermo Fisher Scientific, Waltham, MA, USA) and stored at 4–8 °C overnight prior to being kept at −80 °C before RNA extraction.

### 2.4. Homogenization and Total RNA Extraction

Total RNA was isolated using the ROTI^®^Prep RNA Mini Kit (Carl Roth GmbH & Co. KG, Karlsruhe, Germany) following the manufacturer’s instructions. Tissue homogenization was performed with the Minilys homogenizer (Bertin Technologies SAS, Montigny-le-Bretonneux, France) and ceramic bead tubes (lysis tubes J, Analytik Jena GmbH + Co. KG, Jena, Germany). The homogenization process lasted for 1 min, followed by a 1 min incubation on ice in 100 µL of lysis buffer from the RNA kit. After transferring the supernatant, the samples were treated with DNase I (Invitrogen, Waltham, MA, USA) as an additional step. Elution was conducted with 20 µL of nucleic-acid-free water (Carl Roth GmbH + Co. KG, Karlsruhe, Germany). The quantity and quality of RNA were assessed using a spectrophotometer (Nanodrop, Thermo Fisher Scientific, Waltham, MA, USA), and the samples were subsequently stored at −80 °C.

### 2.5. RNA-Seq of Frozen-Thawed Ovarian Tissue

To evaluate potential gene expression differences in ovarian tissue following cryopreservation, whole-transcriptome RNA analysis (RNA-seq) was performed on biopsies from ovaries with ONT (*n* = 18) compared to those with DF (*n* = 18). The total RNA samples used for 3′-mRNA-seq analyses were quantified by fluorometric measurement using the Qubit device and RNA High Sensitivity assay (Thermo Fisher Scientific Inc., Waltham, MA, USA), and quality was measured by capillary electrophoresis using the Fragment Analyzer and the DNF-471 Total RNA High Sensitivity Assay (Agilent Technologies Inc., Santa Clara, CA, USA). 50 ng of total RNA per sample was used for library preparation performed according to the manufacturer’s protocol using the QuantSeq 3′ mRNA-seq V2 Library Prep Kit with UDI and UMI Second Strand Synthesis Module for QuantSeq FWD (Lexogen GmbH, Vienna, Austria). Bead-purified libraries were normalized and finally sequenced on the NextSeq2000 system (Illumina Inc., San Diego, CA, USA), using single-end sequencing with a read length of 100 base pairs. The bclconvert tool v3.8.4 was used to convert the bcl files to fastq files, as well as for adapter trimming and demultiplexing.

### 2.6. RT-qPCR Analysis of Candidate Gene Expression

For the verification of the results of the RNA-seq, we selected 2 candidate genes, *Histone H2B* and *mitochondrial NADH dehydrogenase subunit 6* (*MT-ND6*), which showed significance (Bonferroni < 0.05 and a fold change (FC) > 1.5). Due to the limited amount of remaining RNA samples after RNA-seq, qPCR validation was performed on *n* = 16 DF vs. *n* = 16 ONT samples. GAPDH and ACTB served as reference genes (geometric mean normalization), validated for the human ovarian cortex using TaqMan assays [20]. cDNA synthesis was performed using the Invitrogen cDNA kit (Invitrogen, Waltham, MA, USA) according to the manufacturer’s instructions in a thermocycler (Mastercycler X40, Eppendorf, Hamburg, Germany). For RT-qPCR, TaqMan assays were used in a multiplex with the master mix for multiplexes (Thermo Fisher Scientific, Waltham, MA, USA) [21]. 0.5 µL cDNA per assay was used. The following TaqMan assays were used: ACTB (Assay ID: Hs016060665_g1), GAPDH (Assay ID: Hs02786624_g1), H2B (Assay ID: Hs00606684_s1), and MT-ND6 (Assay ID: Hs02596879_g1). RT-qPCR was performed and analyzed using QuantStudio 5 (Thermo Fisher Scientific, Waltham, MA, USA) with the protocol according to the manufacturer’s instructions.

### 2.7. Immunohistochemistry of H2B and MT-ND6 in Formalin-Fixed, Paraffin-Embedded Tissue of the Same Frozen-Thawed Ovarian Tissue Used in RNA-Seq and qPCR

After completion of RNA-seq and qPCR analysis, only *n* = 2 DF and *n* = 2 ONT from the cohort studied remained with sufficient tissue for reliable immunohistochemistry for H2B and MT-ND6. After thawing as described in Section 2.2, the biopsies were initially washed for 10 min in distilled water (Aqua dest.) and then fixed overnight at RT in Bouin-Hollande fixation solution (No. 12588, Morphisto GmbH, Offenbach am Main, Germany) instead of pure formalin to enhance immunohistochemistry (IHC) analysis [22]. After this, the samples were washed twice with Aqua dest. for one hour each, and then fixation continued with 10% formalin. The Leica TP1020 was used to process the tissue. First, the tissue was fixed in 10% formalin, followed by a series of ethanol solutions, and finally, it was washed three times in 99% ethanol. The samples were then cleared in xylene three times and infiltrated two times in paraffin for more than 6 h.

The formalin-fixed, paraffin-embedded tissue (FFPE) samples were cut into sections of 3 µm thickness using a Microm HM355 rotary microtome (Thermo Fisher Scientific formerly Microm International GmbH, Walldorf, Germany). After that, the sections were heated on slides. For deparaffinization, the sections were incubated in xylene (Morphisto GmbH) for 20 min. Then, the slides were washed with isopropanol twice following a series of ethanol washes (96%, 80%, and 70% EtOH) and Aqua dest. The sections were also stained using the Azan (azocarmine–aniline blue) method according to Heidenhain (Ref. 12079, Morphisto GmbH), following the manufacturer’s instructions with adjusted incubation times (2 h and 30 min, respectively). Azan staining was applied to achieve enhanced contrast and clear differentiation of ovarian stromal structures, which are not adequately resolved by standard hematoxylin-eosin (H&E) staining.

For antigenic unmasking, a 10 mM citrate buffer with a pH of 6.0 (ZUC028, Zytomed Systems, Berlin, Germany) was used and heated up to 97 °C for 20 min with a Tefal Vitacuise steamer (Groupe Seb WMF Consumer GmbH, Geislingen an der Steige, Germany).

The slides were washed first with Aqua dest., followed by TRIS Wash Buffer B (ZUC066, Zytomed Systems) for 10 min. The tissues were then incubated in blocking solution, Animal-Free Blocker^®^ & Diluent, R.T.U. (Vector Laboratories, Newark, CA, USA), for 60 min. The primary antibodies, H2B (12364, Cell Signaling, 1:1000 dilution) and MT-ND6 (PA5-109993, Invitrogen, 1:100 dilution), were diluted in antibody diluent (ZUC025, Zytomed Systems) and incubated overnight at 4–8 °C. For validation of the antibodies used in IHC, human placenta, human kidney tissues and cerebellum served as positive controls (see Supplementary Figures S1–S4). The visualization of protein expression for H2B and MT-ND6 was conducted using the alkaline phosphatase (AP) polymer kit (POLAP, Zytomed System), following the manufacturer’s instructions. After being incubated with the primary antibody, the sections were washed with wash buffer (ZUC066, Zytomed Systems) and then blocked with Post Block (secondary antibody from POLAP-100, Zytomed Systems). The sections were washed with wash buffer again and then put in the AP for 30 min at RT. After this incubation, the sections were washed again with TRIS Wash Buffer (ZUC052, Zytomed Systems). The permanent AP-Red-Kit (ZUC001, Zytomed Systems) was utilized as a chromogen according to the manufacturer’s protocol.

Counterstaining of the tissue sections was performed using hematoxylin (CATHE-M, Biocare Medical, Pacheco, CA, USA), diluted 1:10 with Aqua dest. and incubated for 10 min. The bluing process was conducted in warm running tap water, followed by a washing step for 10 s in Aqua dest. Finally, the dehydration was done in 96% and 99% isopropanol, clearing in xylene, and covering with glass and ROTI^®^ Histokitt II (Carl Roth GmbH & Co. KG, Karlsruhe, Germany). An Olympus CX43 microscope (Olympus Corporation, Tokyo, Japan) and the Scan & LiveView system from PathoZoom (smart in media AG, Cologne, Germany) were used for examination.

### 2.8. Statistics

For the RNA-seq analysis, the data analyses on FASTQ files were performed with CLC Genomics Workbench (version 22.0.2; QIAGEN, Venlo, The Netherlands). The first UMI tag was removed from all reads and annotated to be read. Reads without UMI were discarded. Afterward, reads of all samples were trimmed for adapter and quality (with default parameters): Bases below Q13 were trimmed from the end of the reads (maximum 2 ambiguous nucleotides). Mapping was performed against the Homo sapiens genome sequence (GRCh38). After grouping the samples according to their respective experimental conditions, group comparisons were performed and statistically analyzed using the CLC-inbuilt RNA-seq analysis workflow. The resulting p-values were corrected for multiple testing using the False discovery rate (FDR) and Bonferroni correction. A *p*-value of <0.05 was considered significant. The ΔΔCt method was used for the relative quantification of gene expression analysis for RT-qPCR. The differences in the Ct values of DF and ONT were analyzed by an unpaired *t*-test with GraphPad Prism 8.0 (GraphPad Software Inc., San Diego, CA, USA).

## 3. Results

### 3.1. Limited Differential Expression Variations in Frozen-Thawed Ovarian Tissue After ONT

**Figure 1:**
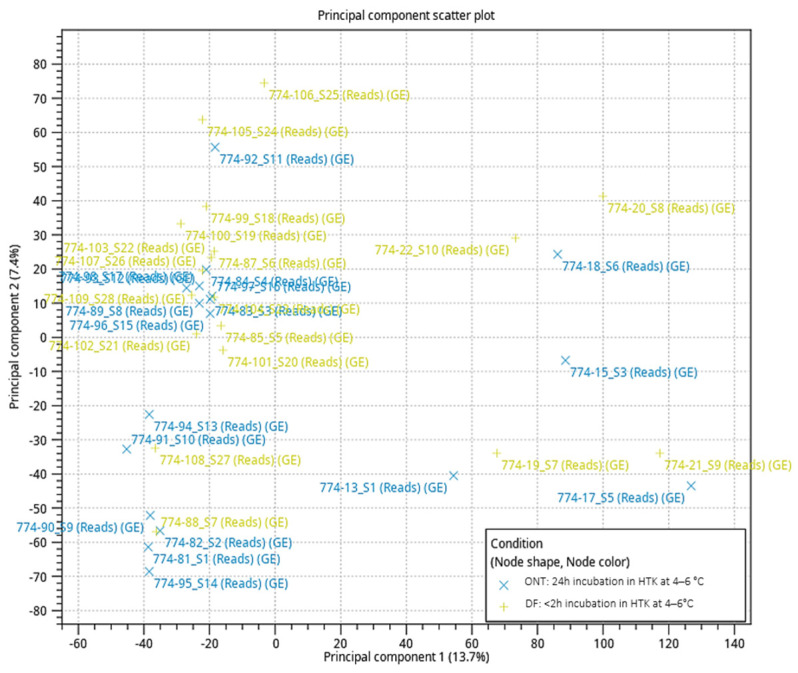
PCA of RNA-seq data derived from frozen-thawed ovarian tissue. Each point stands for one patient (yellow for the DF group and blue for the ONT group). PC1 and PC2 account for 7.4% and 13.7% of the overall variance, respectively. The plot shows that the groups are only partially separated, which suggests that there are only small differences in transcription.

The RNA-seq analysis was performed on 36 frozen-thawed ovarian biopsies. The DF group (*n* = 18) served as the control and was compared to the ONT group (*n* = 18). The Principal Component Analysis (PCA) plot indicates that the DF and ONT groups have a PC1 variance of 7.4% and a PC2 variance of 13.7%. This shows that the global gene expression between ONT and DF appears to be similar (Figure 1). Using the FDR-based primary analysis, 11 genes met the significance threshold (FDR < 0.05, fold change > 1.5; Supplementary Table S2). Of these, 6 genes remained significant after Bonferroni correction (fold change > 1.5), underscoring that only a very small subset of genes shows consistent upregulation between the conditions.

**Figure 2:**
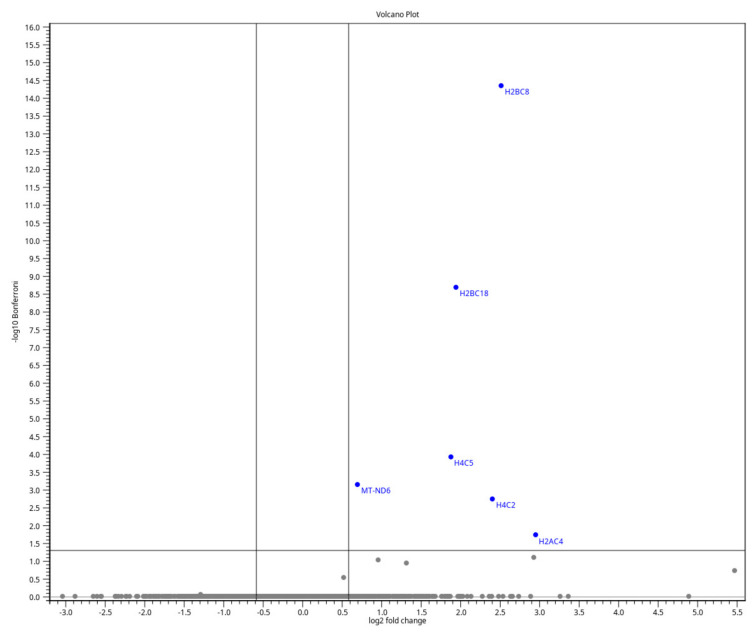
A volcano plot showing differentially expressed genes between frozen-thawed ovarian tissue groups (*n* = 36; 18 DF versus 18 ONT) illustrates the limited number of transcriptional changes identified by RNA-seq. Each point represents a single gene. Significantly regulated genes are highlighted in blue with FC > 1.5 and a Bonferroni-adjusted *p*-value < 0.05 (corresponding to log2FC > 0.585 and −log10 Bonferroni > 1.3).

**Figure 3:**
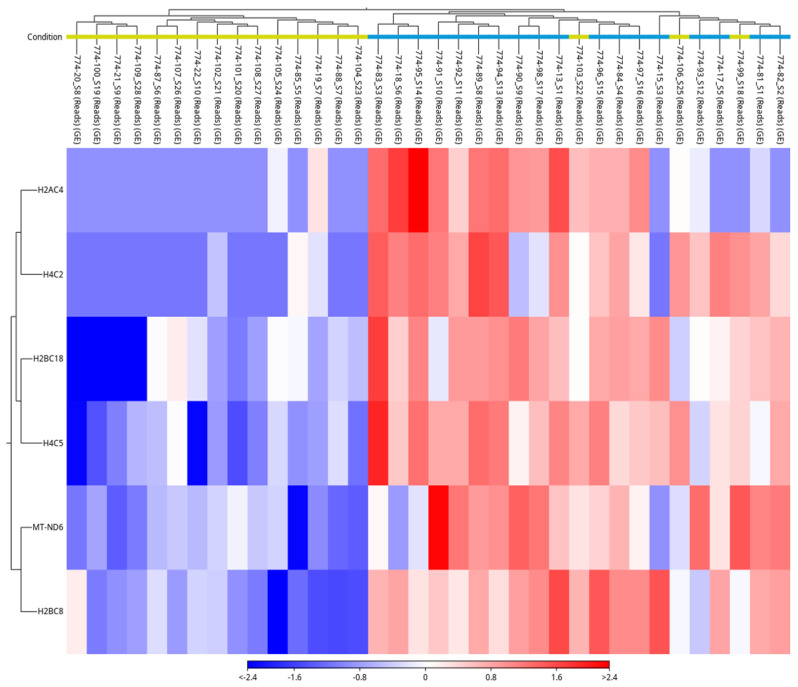
Heatmap showing the expression of differentially expressed genes in frozen-thawed ovarian tissue (*n* = 36; 18 DF versus 18 ONT). Rows represent genes, and columns represent individual samples. Yellow represents the DF group, and blue represents the ONT group. Hierarchical clustering reveals general group-specific expression patterns, although some inter-individual variability is observed.

The upregulated genes in the volcano plot include H2BC8, H2BC18, MT-ND6, H4C5, H4C2, and H2AC4, most of which belong to the histone family (Figure 2). Hierarchical clustering of the samples revealed a general grouping trend in the heat map, although three samples clustered with the opposite group. This overlap suggests inter-individual variability rather than substantial gene expression changes (Figure 3). Overall, these results indicate only a limited number of consistent differences in gene expression within the ovarian tissue with ONT in cooled HTK solution. Due to the small number of detected differentially expressed genes, gene ontology enrichment analysis was not performed.

### 3.2. Validation of Candidate Genes H2B and MT-ND6 in Frozen-Thawed Ovarian Tissue by RT-qPCR

**Figure 4:**
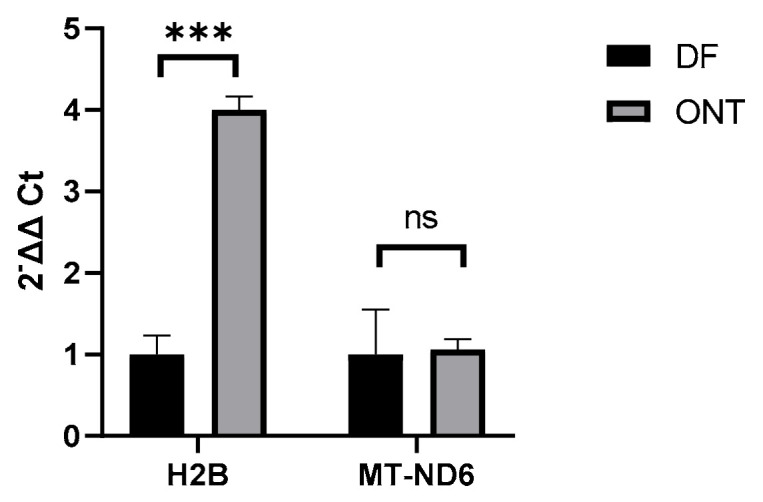
Expression of the candidate genes H2B and MT-ND6 measured by RT-qPCR in the same patient cohort (*n* = 16 DF versus 16 ONT due to insufficient RNA quantity). GAPDH and β-actin were used as reference genes. Gene expression was normalized to DF (see Supplementary Tables S3 and S4). Data are shown as 2ΔΔCt as mean ± SEM, and statistical significance was assessed by an unpaired *t*-test on ΔCt values.*** = *p* < 0.001. Two technical runs were performed.

We validated the RNA-seq findings by RT-qPCR, examining *H2BC8* (log_2_FC 5.73, Bonferroni-adjusted *p* = 4.5 × 10^−15^), *H2BC18* (log_2_FC 3.85, Bonferroni-adjusted *p* = 2.05 × 10^−9^), and *MT-ND6* (log_2_FC 0.7, Bonferroni-adjusted *p* = 0.0007). The TaqMan qPCR primers could only detect total *H2B* expression, without distinguishing subunits. Since core histones are mostly co-regulated and *H2B* had the highest FC, it was taken as a representative marker for the upregulated histones (Figure 2 and Figure 3). Besides the high FC, the candidate genes were chosen because of their biological relevance for chromatin modulation and energy metabolism. *H2B* showed a fourfold increase in gene expression in the ONT group (FC 4.5, *p* < 0.001), which is in line with the observation in RNA-seq (Figure 4). By contrast, *MT-ND6* had an FC of 1.1 and *p* = 0.88 from the unpaired *t*-test and resulted in no significant change in expression of the ONT group (Figure 4).

### 3.3. Spatial Protein Expression of H2B and MT-ND6 in Frozen-Thawed Ovarian Tissue

**Figure 5:**
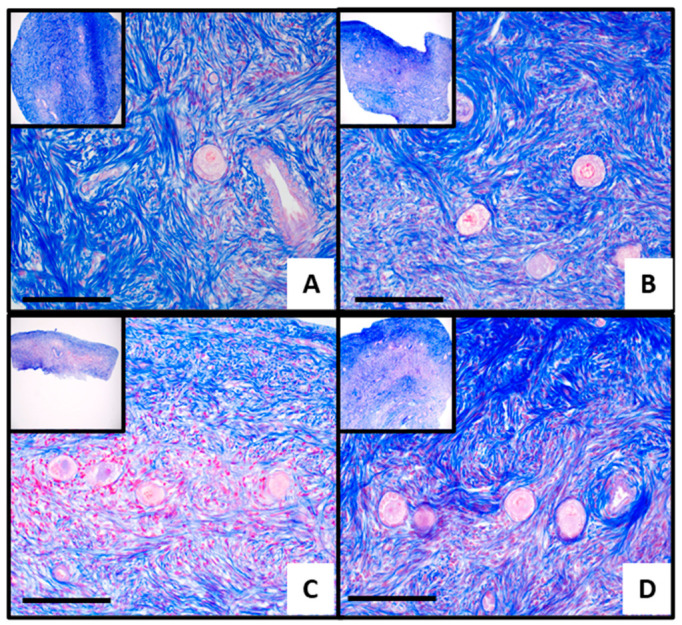
Azan staining of four frozen-thawed ovarian tissues from the DF group (**A**,**B**) and from the ONT group (**C**,**D**). Stromal connective tissue is stained blue, and follicular and other cellular components appear red. Tissue architecture is clearly visible, and cells appear morphologically intact, indicating preserved vitality. Image at 100× and 400× magnification; scale bar = 500 µm.

**Figure 6:**
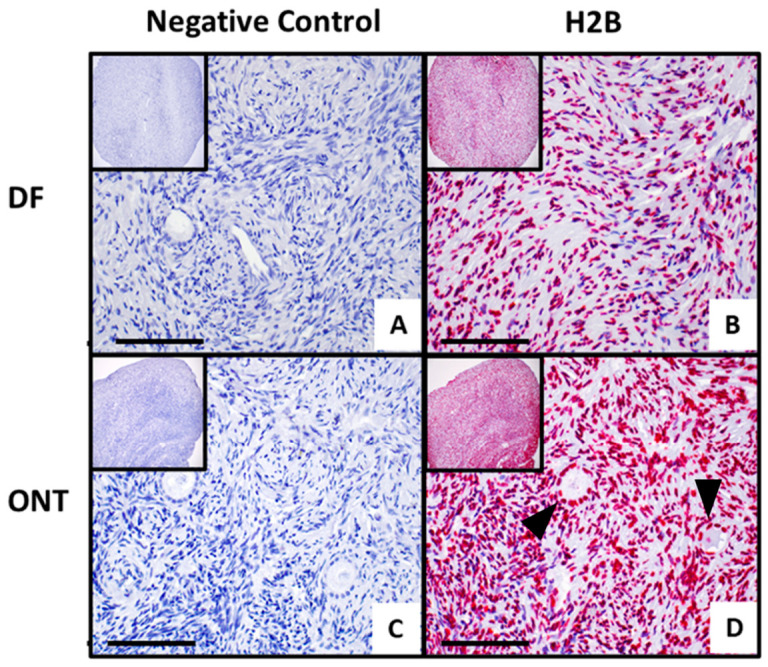
Representative immunohistochemistry of qualitative H2B expression in ovarian tissue with the conditions DF (**A**,**B**) compared to tissue with the condition ONT (**C**,**D**) at 100× and 400× magnification; scale bar = 500 µm. (**A**,**C**) are the negative controls with only antibody diluent. Black arrows indicate follicles.

Azan staining effectively highlighted ovarian tissue structures with high contrast, clearly delineating stromal and follicular regions. The cells and stroma appeared morphologically intact, indicating tissue vitality across all four samples (Figure 5A–D). IHC revealed the presence of H2B and MT-ND6 in FFPE samples from the frozen-thawed ovarian biopsies of the original RNA-seq cohort, which included *n* = 2 DF and *n* = 2 ONT samples. According to the predicted protein expression data from the Human Protein Atlas, H2B exhibited a ubiquitous nuclear expression in ovarian tissue (Figure 6B,D), with notably high levels in follicle cells as well as in the ovarian stroma [23]. In the ONT samples, the H2B expression appeared slightly higher.

**Figure 7:**
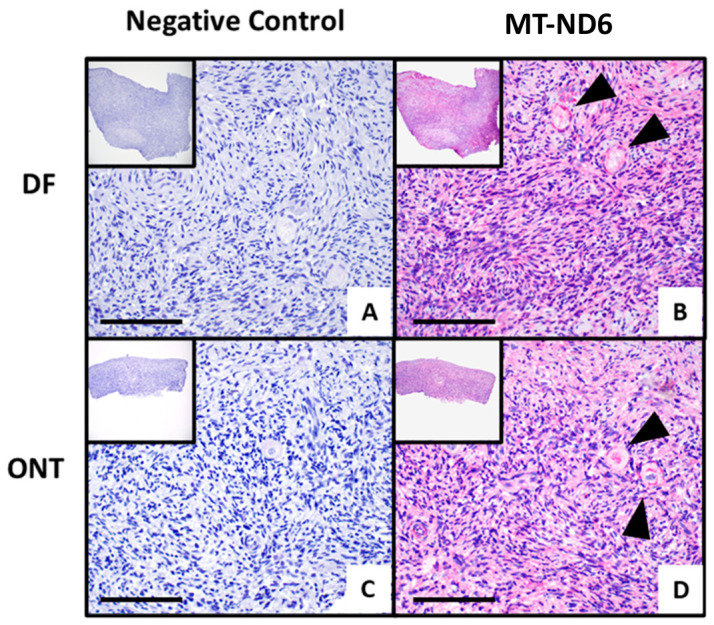
Representative immunohistochemistry of qualitative MT-ND6 expression in ovarian tissue with the condition DF (**A**,**B**) compared to tissue with the condition ONT (**C**,**D**) at 100× and 400× magnification; scale bar = 500 µm. A and C are the corresponding negative controls with only antibody diluent. Black arrows indicate follicles.

For MT-ND6, to date there are no ovarian tissue protein expression profiles, and thus the subcellular location is not available, but the membrane is named as the predicted location [23]. In our ovarian biopsies, we detected a cytoplasmic expression with a granular pattern and a higher expression in the follicles (Figure 7B,D). The MT-ND6 expression pattern appeared similar between the DF and ONT samples. Due to the limited remaining ovarian tissue of the original RNA-seq cohort and its heterogeneity, the comparison of DF versus ONT was considered exploratory.

## 4. Discussion

While clinical pregnancy rates after ONT vs. DF are comparable [9], molecular effects of transport remain largely unexplored. To our knowledge, this RNA-seq study (*n* = 36) is the first to demonstrate transcriptomic stability with only modest histone/MT-ND6 upregulation after 24 h in HTK. Our study provides the first RNA-seq analysis of transport effects in ovarian tissue within the standardized FertiPROTEKT protocol regarding overnight transportation of ovarian tissue prior to cryopreservation. It remains unclear what happens at the molecular level in ovarian tissue both before and after OTC, although a cooled HTK solution is known to preserve the tissue integrity and quality of whole organs, such as the heart or liver [13]. Therefore, this study aimed to identify any potentially differentially expressed genes after 24 h HTK incubation at 4–8 °C, which is the common condition for transporting a patient’s ovary to a centralized cryobank from other clinics. Our study supports previous findings that prolonged storage does not impair ovarian tissue integrity and overall quality, and additionally extends these observations to the transcriptomic level [24]. From a clinical perspective, the preserved molecular homeostasis we observed is consistent with the comparable pregnancy and live birth rates reported by the FertiPROTEKT network [9] and other published data after ONT versus DF, thereby reinforcing the clinical feasibility of decentralized retrieval with centralized cryobanking. Our interpretation aligns with pregnancy and live birth rates reported after ovarian tissue transplantation in the German IVF registry (Deutsches IVF-Register, DIR), which includes data from the FertiPROTEKT network. DIR data represent the consolidated results of the FertiPROTEKT network and thus also include the three largest centralized cryobank institutions cited, which follow identical protocols for transport and slow-freezing procedures. While these registry reports do not differentiate transport conditions and thus cannot demonstrate formal equivalence between overnight transport and direct freezing, the modest RNA changes without stress-related signatures are consistent with non-inferiority trends observed in published FertiPROTEKT cohorts [9].

We acknowledge that our cryopreservation protocol is not globally standardized. However, we deliberately used the modified Gosden slow-freezing protocol recommended as the standard within the FertiPROTEKT network. This network protocol underlies the largest published clinical cohorts and the outcome analysis in the current 2024 German IVF Registry reports, maximizing comparability between our molecular data and the most extensive available clinical outcomes, despite international protocol heterogeneity [8,9,11].

Our study supports the results of previous studies that prolonged storage does not impair ovarian tissue integrity and overall quality and additionally extends these findings to the transcriptomic level [24]. From a clinical perspective, our data support decentralized laparoscopic retrieval with centralized cryobanking, as overnight transport in cooled HTK did not induce detrimental transcriptomic stress signatures. Our transcriptomic stability aligns with FertiPROTEKT/DIR results (no significant ONT vs. DF differences in CPR/LBR) and other morphological data [9,25]. Centralized cryobanking, primarily performed according to FertiPROTEKT standard protocols, can significantly reduce variability in transport, processing and cryopreservation [7,8,9,10,11]. Our study complements the morphological findings on transcriptomic readouts under comparable hypothermic transport conditions, rather than readdressing their already resolved question on primordial follicle integrity thresholds.

Importantly, our study design captures a combined effect of overnight hypothermic transport in HTK solution, which is the standard transport medium in all FertiPROTEKT centralized cryobanking institutions, together with subsequent standardized slow-freezing, rather than an isolated transport intervention. This composite exposure closely mirrors the real-world FertiPROTEKT standard, in which ovaries are transported overnight and then cryopreserved centrally according to harmonized slow-freezing protocols [26]. Therefore, the observed transcriptomic stability may reflect a favorable effect of the clinically implemented workflow and should not necessarily be interpreted as a transport-only effect.

Our transcriptomic data indicate that tissue homeostasis remains mostly balanced after ONT. Analysis of the relatively large groups of *n* = 18 DF and *n* = 18 ONT in the RNA-seq study revealed only small variance in the PCA plot, with PC1 accounting for 7.4% and PC2 for 13.7% (Figure 1). The cumulative PC of only 21.1% indicates high homogeneity between the two groups, suggesting a minimal global biological effect of ONT on ovarian tissue. However, the partial overlap of DF and ONT samples in the PCA plot also reflects inter-individual variability and indicates that subtle group differences cannot be completely ruled out.

Despite no globally significant transcriptional changes in the ONT group, statistical analysis of differentially expressed genes in the ONT group revealed six upregulated genes after stringent Bonferroni correction (Figure 2). FDR–based correction similarly confirmed a very limited impact of ONT, with only eleven genes being upregulated and none downregulated and no enrichment of stress- or apoptosis-related pathways (Supplementary Table S2). Interestingly, most of these genes after correction belonged to the histone family. This accumulation of histone-related genes may suggest the modulation of chromatin pathways after ONT [27,28].

We selected two candidate genes for further analysis by RT-qPCR in the same cohort of frozen-thawed samples used for RNA-seq. The first was *H2B*, as several *H2B* subtypes showed a high log2 fold change in the ONT group (Figure 2). As core histones are primarily co-regulated, *H2B* serves as a representative of upregulated histones. The second was *MT-ND6*, which plays a crucial role in energy production and adaptive stress responses, both of which are influenced by cooled HTK [29].

*H2B* showed a significant increase in qPCR (Figure 4), which was consistent with the RNA-seq results. In contrast, *MT-ND6* expression was stable in the ONT group by qPCR (FC 1.1, *p* = 0.88), differing from the marginal increase (FC 1.5) observed in the RNA-seq data. This discrepancy may stem from the modest FC in RNA-seq, reduced statistical power due to a subset of samples in qPCR, or differences in assay design and sensitivity between the two methods. Thus, *MT-ND6* upregulation in RNA-seq should be interpreted cautiously. The presence and localization of H2B and MT-ND6 proteins were assessed in FFPE ovarian tissue under DF and ONT conditions. AZAN staining clearly showed viable and intact stromal tissue and follicles in all four examined ovaries (Figure 5A–D). H2B was detected—as expected—in cell nuclei, with high expression in the stromal and granulosa cells of primordial follicles [23]. An exploratory comparison (*n* = 2 DF vs. *n* = 2 ONT) suggested an increasing trend in the ONT samples (Figure 6D).

To our knowledge, this is the first study to report the IHC localization of MT-ND6 in human ovarian tissue, as no data are available in the Human Protein Atlas [23]. We observed cytoplasmic expression with a granular pattern in the stromal cells and oocytes of the follicles (Figure 7B,D), with no major differences between the ONT and DF groups in the representative staining (Figure 7D).

Although RNA-seq showed no global molecular changes in the ONT group, individual genes appeared to be affected. H2B upregulation confirmed by qPCR was mirrored by a slightly higher protein expression, which in this exploratory IHC set might reflect a specific effect of ONT. H2B is a part of nucleosomes around which DNA wraps to form chromatin and chromosomes, enabling tight packaging into the nucleus [30]. Increased H2B expression is linked to cellular responses in chromatin-associated stress pathways, contributing to genomic integrity and stability [31,32]. Specific modifications, such as H2B monoubiquitination, play a role in DNA repair and require further investigation [33].

*MT-ND6* mRNA was slightly higher in ONT by RNA-seq but stable by qPCR, consistent with IHC (Figure 7D). As a key subunit of Complex I (NADH dehydrogenase) in the electron transport chain (ETC), *MT-ND6*, encoded by mtDNA, transfers electrons from NADH to ubiquinone for energy generation and stabilizes the complex [34]. Located on the inner mitochondrial membrane, it is the main ROS source during ETC activity and is critical for energy balance and oxidative stress responses after ischemia [35]. Our focus was on *MT-ND6*, as post-retrieval ovarian tissue undergoes ischemia, impairing the ETC and severely reducing energy production [36]. Prolonged hypoxia and ROS accumulation can trigger follicle apoptosis, especially after transplantation and reperfusion injury [37]. HTK reduces metabolism and ROS-mediated damage; cooling in this solution preserves low-level metabolism and limits ROS buildup [15,38]. Sustaining minimal *MT-ND6* expression may stabilize Complex I, supporting residual ATP production under nutrient-limited conditions. A recent study showed that MT-ND6 reduction causes mitochondrial dysfunction in lymphocytes and promotes inflammation [39].

This study has some limitations. First, we did not include a fresh, non-transported, non-cryopreserved control of ovarian cortex, so we cannot disentangle transcriptional effects of overnight transport from those induced by the freezing–thawing procedure itself. Consequently, the observed transcriptomic stability and modest gene expression changes may reflect the combined impact of cooling in HTK and cryopreservation rather than transport alone.

Our results suggest that *H2B* is upregulated and *MT-ND6* is stably expressed in ovarian tissue after ≤24 h of cooling with HTK compared to a maximum of ≤2 h post-retrieval, potentially aiding homeostasis. Most RNA-seq genes showed no significant changes between the ONT and DF groups, indicating overall transcriptome stability without evidence of apoptosis or stress pathways that could damage tissue or follicles. However, RNA-seq misses some low-abundance mRNA isoforms, and stable mRNA does not rule out translational differences; subtle protein-level changes may occur despite no mRNA shifts [40,41,42]. Future downstream analyses (e.g., proteomics, Western blotting, and mass spectrometry), miRNA or splicing effects, and further pre-cryopreservation biopsies could provide a more comprehensive understanding of the effects of ONT on ovarian tissue.

## 5. Limitations

This study has several limitations. First, it is a single-center mechanistic analysis with a relatively small sample size (*n* = 18 per group), which limits statistical power and the generalizability of our findings. Second, our design captures a combined exposure of hypothermic transport and slow-freezing within one specific workflow so that isolated effects of transport duration or alternative cryopreservation protocols cannot be distinguished. Third, clinical outcome data from network-based registries are used only as contextual background and cannot be directly linked to the transcriptomic results due to residual confounding and differences in patient selection and center practices. Further limitations are that we did not include a fresh, non-transported, non-cryopreserved control of ovarian cortex, so we cannot disentangle transcriptional effects of overnight transport from those induced by the freezing–thawing procedure itself. Consequently, the observed transcriptomic stability and modest gene expression changes may reflect the combined impact of cooling in HTK and cryopreservation rather than transport alone.

## 6. Conclusions

In summary, our findings indicate that throughout ONT, ovarian tissue homeostasis is mostly maintained when cooled in HTK solution prior to cryopreservation. Additionally, our analysis revealed no increase in apoptotic or tissue-damage-related genes in the frozen-thawed tissues RNA-seq. Only 6 genes (Bonferroni < 0.05, FC > 1.5) were upregulated in the ONT group, with a majority belonging to the histone family. This increase in histone-associated genes indicates a possible modulation of chromatin pathways, and the single mitochondrial gene suggests involvement in metabolic functions for maintaining energy production.

We also detected H2B and MT-ND6 using qPCR and IHC in the same cohort of remaining frozen-thawed samples. The observed increase in H2B, along with the stable expression of MT-ND6, may contribute to maintaining the stability and integrity of ovarian tissue within the ONT group. To our knowledge, we present the first IHC expression pattern of the MT-ND6 protein in human ovarian tissue. Further studies are needed to examine the influence of H2B and MT-ND6 on the viability of ovarian tissue prior to cryopreservation by protein analyses. These findings are consistent with the feasibility of the workflow under the studied conditions of centralized ovarian tissue cryobanking using overnight cooled HTK transportation followed by standardized slow-freezing, reflecting the real-world FertiPROTEKT protocol rather than an isolated transport experiment.

## Data Availability

The original contributions presented in this study are included in the article/Supplementary Material. Further inquiries can be directed to the corresponding author.

## Supplementary Materials

The following supporting information can be downloaded at https://www.mdpi.com/article/10.3390/jcm15093260/s1. Table S1: Baseline characteristics of the included patients. *p*-values from independent samples *t*-tests; Table S2: The 11 significant differentially expressed genes in *n* = 18 DF versus *n* = 18 ONT of ovarian tissue filtered by False Discovery Rate (FDR) < 0.05 and 6 genes are significant differentially expressed genes in with Bonferroni > 0.05; Table S3: Calculation of relative expression of H2B with Δ ct Values of DF and ONT of *n* = 16 of ovarian tissue; Table S4: Calculation of relative expression of MT-ND6 with Δ ct Values of DF and ONT of *n* = 16 of ovarian tissue; Figure S1: Human placenta as positive control for validation of H2B with dilution 1:1000. Shown protein expression in (A) decidual cells and (B) trophoblast cells; Figure S2: Human kidney as positive control for validation of H2B with dilution 1:1000. Shown protein expression in (A) glomeruli and (B) renal tubes in the cortex; Figure S3: Human placenta as positive control for validation of MT-ND6 with dilution 1:1000. Shown protein expression in (A) decidual cells and (B) trophoblast cells. Images at 100× and 400× magnification; Figure S4: Positive controls for validation of MT-ND6 with dilution 1:100, (A) human kidney, (B) cerebellum.

## Abbreviations

The following abbreviations are used in this manuscript:

ONT	Overnight transport
DF	Direct frozen
OTC	Ovarian tissue cryopreservation
RNA-seq	RNA Sequencing
HTK	Histidine-tryptophan-ketoglutarate
PC	Principal Component
PCA	Principal component analysis
NADH	Nicotinamide adenine dinucleotide
MT-ND6	Mitochondrial NADH dehydrogenase subunit 6
ETC	Electron transport chain
FC	Fold change
IHC	Immunohistochemistry
