# Supplementary material for: Transport Time Does Not Substantially Alter RNA Expression in Human Ovarian Tissue After Standardized Slow-Freezing for Fertility Preservation
Source: J Clin Med. 2026 Apr 24;15(9):3260. doi: 10.3390/jcm15093260 (PMC13164559; doi:10.3390/jcm15093260)
Supplement: Supplementary file 1 [file jcm-15-03260-s001.zip › jcm-4234023-supplementary.pdf]

Supplementary Material

Table S1 Baseline characteristics of the included patients. p-values from independent samples t-tests.

Characteristics	ONT			DF			p-value
	N	Mean ± SD	Range	N	Mean ± SD	Range	
Age (years)	18	31.3 ± 2.7		18	29.6 ± 4.5		p=0.18
Minimum age			25			21	
Maximum age			35			35	
Anti-Müllerian Hormone (AMH) (ng/mL)	18	3.0 ± 1.7		18	3.1 ± 1.6		p=0.86
AMH < 1.0 ng/mL, n (%)	3	0.7 ± 0.2		0	/		
AMH 1.0-3.5 ng/mL, n (%)	7	2.2 ± 0.7		9	1.9 ± 0.7		
AMH > 3.5 ng/mL, n (%)	5	4.8 ± 0.09		7	4.7 ± 0.08		
no AMH available, n (%)	3	n.a.		2	n.a.		
Follicular density (per 3x2mm biopsy)	18	90.78 ± 64.1		18	60.1 ± 50.2		p=0.22
Minimum density			19			11	
Maximum density			226			200	
Diagnosis							
Breast cancer, n (%)	18	100%		18	100%		

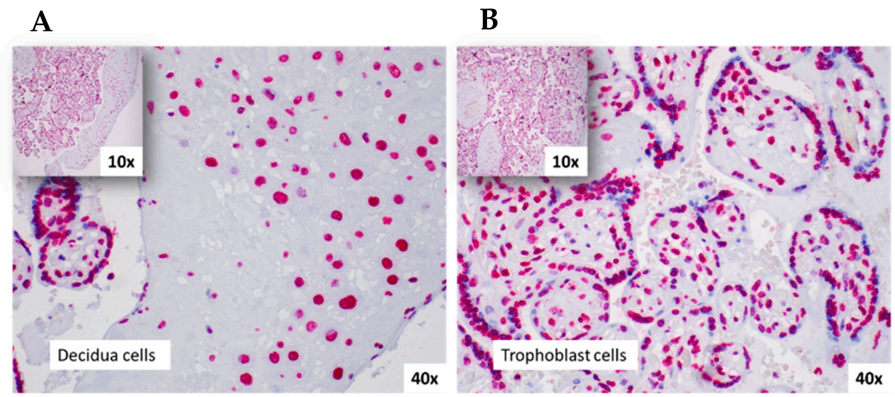

Figure S1. Human placenta as positive control for validation of H2B with dilution 1:1000. Shown protein expression in (A) decidual cells and (B) throphoblast cells. Images at 100x and 400x magnification.

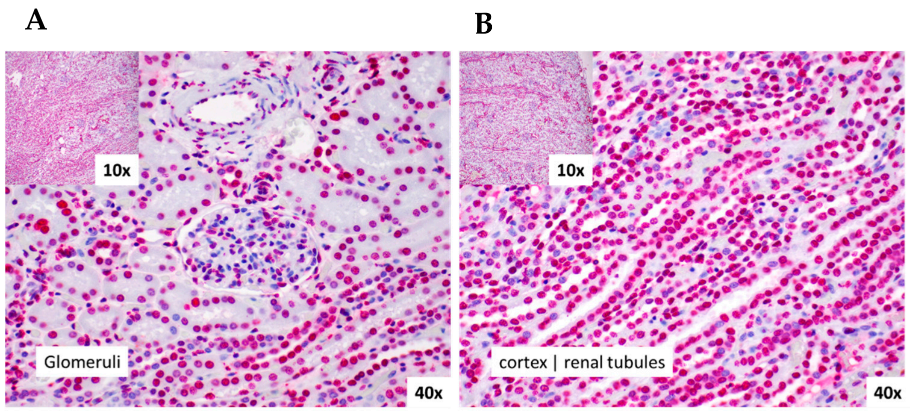

Figure S2. Human kidney as positive control for validation of H2B with dilution 1:1000. Shown protein expression in (A) glomeruli and (B) renal tubes in the cortex. Images at 100x and 400x magnification.

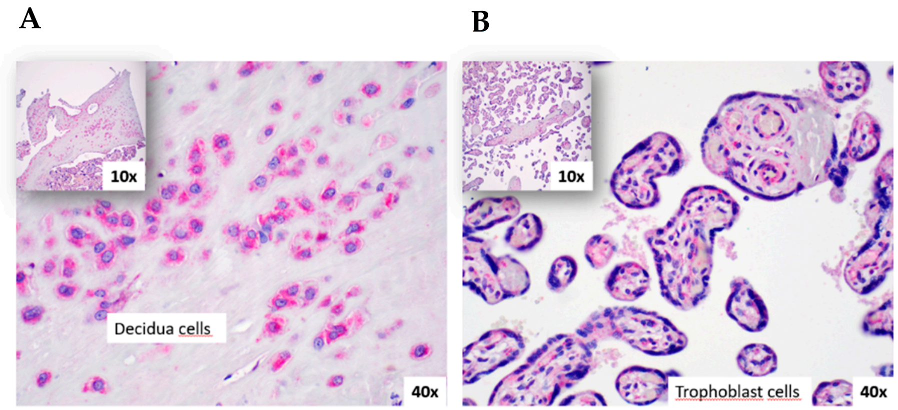

Figure S3. Human placenta as positive control for validation of MT-ND6 with dilution 1:1000. Shown protein expression in (A) decidual cells and (B) throphoblast cells. Images at 100x and 400x magnification.

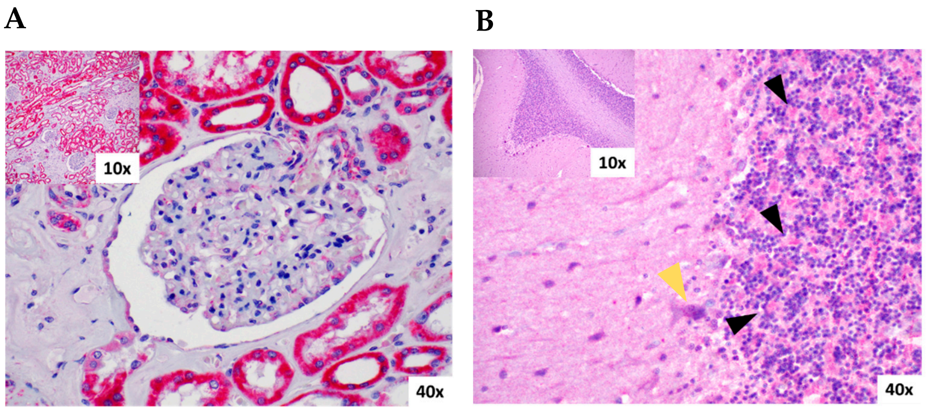

Figure S4. Positive controls for validation of MT-ND6 with dilution 1:100, (A) human kidney, (B) cerebellum. Black arrows indicate the stratum moleculare (outer molecular layer of the cerebellar cortex), and the yellow arrow indicates the Purkinje cell in the stratum purkinjense layer between molecular and granular layer, as in the MTND6 antibody datasheet.

Table S2. The 11 significant differentially expressed genes in n=18 DF versus n=18 ONT of ovarian tissue filtered by False Discovery Rate (FDR) <0.05 and 6 genes are significant differentially expressed genes in with Bonferroni >0.05.

Name	Chromosome	Identifier	Cryopreservation 24 h after operation vs. Cryopreservation directly after operation - Max group means	Cryopreservation 24 h after operation vs. Cryopreservation directly after operation - Fold change	Cryopreservation 24 h after operation vs. Cryopreservation directly after operation - Log fold change	Cryopreservation 24 h after operation vs. Cryopreservation directly after operation - P-value	Cryopreservation 24 h after operation vs. Cryopreservation directly after operation - FDR p-value	Cryopreservation 24 h after operation vs. Cryopreservation directly after operation - Bonferroni
H2AC4	6	ENSG00000278463	2,66667056	7,74104533	2,9525284	1,02495E-06	0,00306923	0,01841535
H2BC3	6	ENSG00000276410	2,68877836	7,60490751	2,9269307	4,34318E-06	0,0111477	0,07803391
H2AC19	1	ENSG00000272196	4,70167314	44,2809623	5,46861467	1,01866E-05	0,01830225	0,18302249
H4C2	6	ENSG00000278705	5,25167145	5,30986354	2,40867479	1,00589E-07	0,00036146	0,00180728
H2BC18	1	ENSG00000203814	13,9977882	3,84973628	1,94475962	1,14031E-13	1,0244E-09	2,0488E-09
H4C5	6	ENSG00000276966	16,0512832	3,67476871	1,87765345	6,61002E-09	3,9587E-05	0,00011876
ADAMTS18	16	ENSG00000140873	20,060778	2,49055658	1,31646818	6,2172E-06	0,0124116	0,11170442
H2BC21	1	ENSG00000184678	23,4929617	1,94636524	0,96078246	5,15179E-06	0,01157027	0,0925622
H2BC8	6	ENSG00000273802	25,484571	5,73620796	2,52009733	2,50981E-19	4,5094E-15	4,5094E-15
MT-ND6	MT	ENSG00000198695	77,0720098	1,62226478	0,69800931	3,9528E-08	0,00017755	0,0007102
BCAT1	12	ENSG00000060982	119,883352	1,43632298	0,5223802	1,61578E-05	0,0263915	0,29030649

Table S3. Calculation of relative expression of H2B with Δ ct Values of DF and ONT of n=16 of ovarian tissue.

Δ ct H2B DF	Δ ct H2B ONT	Mean Δ ct H2B DF	Δ ct H2B ONT	Δ Δct H2B	2^-Δ Δct H2B
3,73	0,27	3,42	1,24	-2,18	4,53
2,90	1,54				
3,30	1,67				
4,54	1,38				
4,06	1,30				
3,37	2,28				
1,61	-0,01				
2,99	1,44				
2,52	1,53				
4,24	1,19				
4,42	0,88				
4,00	1,39				
2,68	1,58				
2,15	0,39				
3,18	0,65				
4,99	2,34				

Table S4. Calculation of relative expression of MT-ND6 with Δ ct Values of DF and ONT of n=16 of ovarian tissue.

Δ ct mtND6 DF	Δ ct mtND6 ONT	Mean Δ ct MT-ND6 DF	Δ ct MT-ND6 ONT	Δ Δct MT-ND6	2^-Δ Δct MT-ND6
-5,3	-4,7	-3,5	-3,6	-0,1	1,1
-5,5	-3,4				
-4,2	-3,2				
-4,0	-2,8				
-4,0	-2,7				
-3,9	-3,7				
-3,8	-3,8				
-4,4	-3,6				
-4,2	-4,1				
-3,1	-3,5				
-3,7	-4,1				
-3,5	-3,2				
-3,9	-3,4				
-3,8	-3,7				
-3,6	-4,0				
4,5	-4,1				
